# Comprehensive characterization of chorionic villi-derived mesenchymal stromal cells from human placenta

**DOI:** 10.1186/s13287-017-0757-1

**Published:** 2018-02-05

**Authors:** Mónica S. Ventura Ferreira, Michaela Bienert, Katrin Müller, Björn Rath, Tamme Goecke, Christian Opländer, Till Braunschweig, Petra Mela, Tim H. Brümmendorf, Fabian Beier, Sabine Neuss

**Affiliations:** 1 Institute of Pathology, RWTH Aachen University, Aachen, Germany; 2 Department of Hematology, Oncology, Hemostaseology and Stem Cell Transplantation, RWTH Aachen University, Aachen, Germany; 3 Helmholtz Institute for Biomedical Engineering, Biointerface Group, RWTH Aachen University, Aachen, Germany; 4 Department of Orthopedic Surgery, RWTH Aachen University, Aachen, Germany; 5 Department for Gynecology, RWTH Aachen University, Aachen, Germany; 6 Department of Translational Wound Research, Centre for Biomedical Education and Research (ZBAF), Witten/Herdecke University, Witten, Germany; 7 Department of Tissue Engineering and Textile Implants, Institute of Applied Medical Engineering, Helmholtz Institute, RWTH Aachen University, Aachen, Germany

## Abstract

**Background:**

Studies in which mesenchymal stromal cells (MSC) from the placenta are compared with multiple MSC types from other sources are rare. The chorionic plate of the human placenta is mainly composed of fetal blood vessels embedded in fetal stroma tissue, lined by trophoblastic cells and organized into chorionic villi (CV) structures.

**Methods:**

We comprehensively characterized human MSC collected from postnatal human chorionic villi of placenta (CV-MSC) by analyzing their growth and proliferation potential, differentiation, immunophenotype, extracellular matrix production, telomere length, aging phenotype, and plasticity.

**Results:**

Immunophenotypic characterization of CV-MSC confirmed the typical MSC marker expression as defined by the International Society for Cellular Therapy. The surface marker profile was consistent with increased potential for proliferation, vascular localization, and early myogenic marker expression. CV-MSC retained multilineage differentiation potential and extracellular matrix remodeling properties. They have undergone reduced telomere loss and delayed onset of cellular senescence as they aged in vitro compared to three other MSC sources. We present evidence that increased human telomerase reverse transcriptase gene expression could not explain the exceptional telomere maintenance and senescence onset delay in cultured CV-MSC. Our in-vitro tumorigenesis detection assay suggests that CV-MSC are not prone to undergo malignant transformation during long-term in-vitro culture. Besides SOX2 expression, no other pluripotency features were observed in early and late passages of CV-MSC.

**Conclusions:**

Our work brings forward two remarkable characteristics of CV-MSC, the first being their extended life span as a result of delayed replicative senescence and the second being a delayed aged phenotype characterized by improved telomere length maintenance. MSC from human placenta are very attractive candidates for stem cell-based therapy applications.

**Electronic supplementary material:**

The online version of this article (doi:10.1186/s13287-017-0757-1) contains supplementary material, which is available to authorized users.

## Background

The human placenta is a highly specialized pregnancy organ for supporting the development of a fetus. It connects the developing fetus to the wall of the mothers’ uterus through the umbilical cord (UC). Although the placenta originally develops from cells of fetal origin, it later consists of both maternal tissue (decidua) and fetal tissue (chorion, aminon). The chorion composition mainly consists of fetal blood vessels embedded in fetal stroma tissue and trophoblastic cells organized into ramified structures called chorionic villi (CV).

More than 10 years ago, researchers introduced the idea of using the placenta as a source for both maternal and fetal mesenchymal stromal cells (MSC) and progenitor cells [1–3]. Later, in 2007, the first international workshop on placenta-derived stem cells took place in Brescia, Italy, with the intention of setting criteria for defining MSC from human placenta [4]. However, a consensus has not yet been reached within the scientific community, as evidenced by the variety of studies published after the 2007 workshop which did not make use of the proposed criteria.

MSC from human placenta differ not only in terminology but also in harvesting and isolation methods [3, 5–20]. Studies comparing MSC from placenta with those from other sources exist, but comparative studies between CV-MSC and multiple MSC types (from other sources) are less frequent in the literature [6, 7, 21–23]. Meanwhile, early preclinical work using CV-MSC for tissue engineering applications has already started in different animal models [24–28].

It is unanimous that the use of both maternal-derived and fetal-derived MSC includes a few advantages [29–32]: noninvasive collection; no ethical concerns, often discarded as medical waste; and attractive immunological properties for allogeneic transplantations. MSC of fetal origin are particularly interesting due to their potential use for autologous applications considering the possibility for prenatal harvest and storage [33]. The possibility of MSC of fetal origin displaying a partial embryonic phenotype [34] is controversially discussed, although it could be a potential additional advantage.

In our study, we comprehensively characterize human chorionic villi-derived MSC (CV-MSC) collected from mature postnatal human placenta by analyzing their potential for growth and proliferation, differentiation, immunophenotype, extracellular matrix (ECM) production, telomere length and aging phenotype, and plasticity. A systematic comparison of CV-MSC with respective counterparts isolated from the bone marrow (BM), adipose tissue (AT), and Wharton’s jelly of the UC is presented.

## Methods

### Isolation and culture of human chorionic villi-derived mesenchymal stem cells

**Fig. 1:**
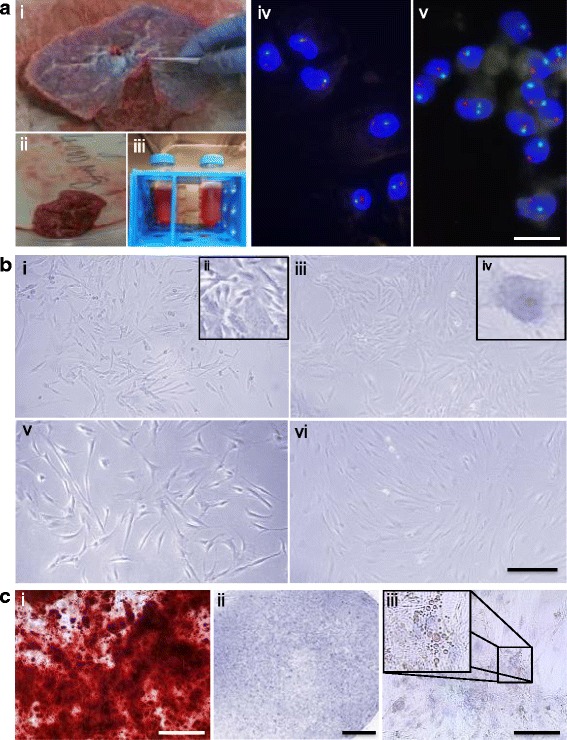
**a** Isolation of human chorionic villi-derived mesenchymal stem cells (CV-CMSC) from human placenta. (i) Removal of the amnion to access the chorionic plate, (ii) pieces of CV dissected from human placenta, (iii) collagenase digestion of CV, and (iv, v) representative images of a CV-MSC donor at passage 4 (iv) and passage 9 (v) after interphase FISH analysis using a X/Y dual color probe. X chromosome fluorescence in green, Y chromosome fluorescence in orange. Scale = 25 μm. **b** Bright-field microscopy images of cultured CV-MSC (i –iv) and BM-MSC (v–vi) right after isolation and plastic adherence (ii), in passage 1 (i, v), in passage 5 (iii, iv), and in passage 10 (vi). Scale = 500 μm. **c** Representative images of Alizarin Red (calcium deposits), Toluin Blue (proteogyclans), and Oil Red O (lipid droplets) stained CV-MSC in passage 3 after respective osteogenic (i, scale = 500 μm), chondrogenic (ii, scale = 1 mm), and adipogenic (iii, scale = 100 μm) differentiation

Placentas from male newborns were collected after cesarean sections at the Department of Gynecology, University Hospital Aachen, in accordance with the local ethical regulations after obtaining informed consent (EK187/08). Placentas were collected from newborns delivered by elective cesarean section. Preterm birth was an exclusion criterion in our study as emergency cesareans were excluded. No further clinical data are available due to the anonymity of the donation. Before the placental tissue was dissected, the fresh placentas were washed extensively with PBS and the maternal decidua portion—identified by an experienced pathologist—was removed. CV-MSC were isolated by digestion. In brief, pieces of approximately 1 cm^3^ equivalent to 4 g (wet weight) were dissected from the CV of the fetal side. Pieces were washed with PBS and transferred to centrifugation tubes for 60-minute digestion with 1 mg/ml Collagenase A (Roche Diagnostics, Germany) at 37 °C (Fig. 1a i–iii). Tissue recovery per placenta was maximized to an average of 200 g (wet weight). Digested tissue was then centrifuged and resuspended in trypsin/EDTA (PAN Biotech, Germany) for 10-minute incubation at 37 °C. Trypsinized tissue was centrifuged and transferred into a single T-25 cell culture flask containing 5 ml Bio-AMF-1 medium including supplements (Biological Industries, Israel) with additional penicillin–streptomycin at 1% and 5 mM l-Glutamine (both Thermo Fisher Scientific, Germany). Cells were kept in a humidified atmosphere at 37 °C with 5% CO_2_ and passaged when 90% confluence was reached.

### Isolation and culture of human bone marrow-derived mesenchymal stem cells

BM-MSC were isolated from bone marrow femoral heads following patient informed consent approved by the local Ethical Committee of the RWTH Aachen University (EK300/13). BM-MSC were isolated as described previously [35] and maintained in supplemented Mesenpan (PAN Biotech, Germany) with 2% fetal calf serum (FCS), 5 mM l-Glutamine and penicillin–streptomycin at 1% (all Thermo Fisher Scientific, Germany).

### Isolation and culture of human umbilical cord-derived mesenchymal stem cells

UC-MSC were isolated from the Wharton’s jelly of UCs as described previously [36]. Tissue collection was performed following local ethical guidelines and receiving informed consent (EK178/08). The cells were maintained in the same type of culture medium as described for BM-MSC.

### Isolation and culture of human adipose tissue-derived mesenchymal stem cells

AT-MSC were isolated from lipoaspirates at the Department of Trauma and Hand Surgery, Medical Faculty of the Heinrich-Heine-University Düsseldorf, according to local ethics guidelines (EK-Nr. 3634). Cell isolation was carried out as described previously [37]. The lipoaspirate was first purified by centrifugation with saline solution, and subsequently by a second centrifugation for 10 minutes at 300 × *g* in order to concentrate the cell pellet before a digestion step with 0.075% collagenase I (Biochrom, Berlin, Germany) for 45 minutes at 37 °C. The digested solution was filtered through a 250-μm filter, and the pellet was concentrated and washed with saline, before being resuspended in culture medium consisting of DMEM/F12 supplemented with 1% penicillin–streptomycin, 10% FCS (all Thermo Fisher Scientific, Germany), and 10 ng/ml bFGF (Peprotech, Germany). A panel of markers including CD16, CD31, CD49d, CD13, and CD29 was used for immunophenotypic characterization of the AT-MSC by flow cytometry (Table 1).

**Table 1:** Antibodies used for flow cytometry, immunofluorescence, and western blot analysis

Flow cytometry	Source	Immunofluorescence	Source
CD44-PeCy7	560569, BD Pharmingen	SM22a	ab10135, Abcam
CD73	550256, BD Pharmingen	Calpomin	Ab700, Abcam
CD105	555690, BD Pharmingen	SM-MHC	M7786, Sigma
HLA-ABC-APC	562006, BD Pharmingen	*α*-SMA	A2547, Sigma
CD34-PerCP-Cy5.5	347222, BD Pharmingen	Tert (1:50)	AP33476PU-N, Acris
CD45-APC	555485, BD Pharmingen	Sox2	PA1-16968, Thermo
CD49a	559594, BD Pharmingen	Nanog	PA1-097X, Thermo
CD146-PE	561013, BD Pharmingen	Oct3/4	sc-5279, Santa Cruz Biotechnology
CD166-PE	559263, BD Pharmingen	Western blot assay	Source
CD19	555410, BD Pharmingen		
CD56-PeCy7	557747, BD Pharmingen	Tert(1:1000)	SAB4502945, Sigma
CD80-PE	560925, BD Pharmingen	Sox2 (1:2000)	PA1-16968, Thermo
CD83-PeCy7	561132, BD Pharmingen	Oct3/4(1:1000)	sc-5273, Santa Cruz Biotechnology
CD86-V450	560359, BD Pharmingen	Nanog (1:2000)	PA1-097X, Thermo
Strol	14-6688-80, eBiosciences	Erk1/2 (1:1000)	9102, Cell Signaling
CD106-PerCPCy5.5	45-0149-42, eBiosciences	pErk1/2(1:1000)	9101, Cell Signaling
CD14-PerCPCy5.5	45-0149-42, eBiosciences	β-Catenin (1:5000)	ab32572, Abcam
CD40-PeCy7	25-0409-41, eBiosciences	Smad2/3 (1:400)	AF3797, R&D Systems
CD275(B7H2)-PE	12-5889-41, eBiosciences	pSmad2/3(1:1000)	8828, Cell Signaling
CD90-APC	17-0909-42, eBiosciences	NICD(1:500)	ab8925, Abcam
HLA-DR-efluor450	48-9956-41, eBiosciences	Akt1/2(1:1000)	sc-1619, Santa Cruz Biotechnology
α-SMA	A2547, Sigma	pArk1/2(1:1000)	9271, Cell Signaling
SM22α	ab10135, Abcam	Smad1/5/9(1:2000)	ab66737, Abcam
vWF	ab8822, Abcam	PSmad1/5(1:1000)	9516, Cell Signaling

All antibodies were used in 1:100 dilutions, unless otherwise indicated

### Sex chromosome detection by fluorescence in-situ hybridization

To confirm the fetal origin of the CV-MSC isolated from placentas collected after the birth of male newborns, X/Y chromosome analysis was performed at early (p3–p4) and late (p8–p10) passages for all donors used for different subsequent experiments (*n* = 5) (Fig. 1a iv–v). The ZytoLight CEN X/Y Dual Color Probe (Zytomed Systems, Germany) was used for detection of human α-satellites of X and Y chromosomes by fluorescence in-situ hybridization (FISH). Ten microliters of the hybridization mixture was added to the cytospun cells, and DNA denaturation was then performed at 75 °C for 2 minutes with subsequent overnight incubation using a humidified chamber. After hybridization, the cytospins were washed with cytology stringency buffer for 2 minutes at 72 °C and subsequently rinsed in sodium chloride/sodium citrate buffer for 1 minute at room temperature (RT). Slides were finalized using Vectashield antifade mounting medium containing 4′,6-diamidino-2-phenylindole (DAPI) (Vector Labs, Germany) and fluorescence detected on an Axiovert S135 microscope (Zeiss, Germany). The α-satellite sequences of the centromere of chromosome X were excited at 488 nm and of chromosome Y at 554 nm.

### Multilineage differentiation

MSC differentiation protocols were applied as described in detail previously [35]. For adipogenic differentiation we used adipogenic induction and maintenance medium [35], alternately, twice a week for 21 days, before cells were fixed with 50% ice-cold ethanol and stained with 0.2% Oil Red O solution (Sigma, Germany) for lipid visualization. For chondrogenic differentiation, pellet cultures were performed and maintained for 21 days in serum-free chondrocyte induction medium refreshed three times a week (with freshly added TGF-β_3_). Pellets were fixed in formalin and embedded in 3% agarose for paraffin block preparation. Slices were prepared and stained with 1% Toluidin Blue (Sigma, Germany) for proteogyclan visualization. For osteogenic differentiation, we applied osteogenic induction medium for 21 days, refreshed three times per week. Finally cells were fixed with 70% ice-cold ethanol and stained with 40 mM Alizarin Red (Sigma, Germany) solution for calcium deposit visualization. Staining was photographed with a Cool Snap™ HQ2 digital camera (Photometrics, USA) on an Axiophot 2 microscope (Carl Zeiss, Germany).

### Long-term cultures

During long-term cultures, cell population-doubling (PD) levels were assessed by manual counting, using Neubauer chambers. In order to standardize reporting of cellular aging, we calculated cumulative PD (cPD) after each passage by applying the following formulas:

$$ {2}^{\mathrm{PD}}=\mathrm{nunmber}\ \mathrm{of}\ \mathrm{harvested}\ \mathrm{cells}/\mathrm{number}\ \mathrm{of}\ \mathrm{seeded}\ \mathrm{cells},\mathrm{cPD}={\Sigma}^{{\mathrm{n}}_2}\left( PD\kern0.24em 1+ PD\kern0.28em 2+\dots + PD\kern0.24em n\right). $$

MSC from all sources were seeded at a density of 5000 cells per cm^2^ in T75 culture flasks. Cell morphology was visualized using a Cool Snap™ HQ2 digital camera (Photometrics, USA) on an Axiophot 2 microscope (Carl Zeiss, Germany).

### Cell viability assays

To assess cell viability, we used the CellTiter-Blue Assay (Promega, Germany) as described previously [38]. MSC in passage 2 were seeded at 5 × 10^4^ cells per well in 96-well plates. Viability was measured after initial cell adhesion to the culture plastic and again after 7 days in culture with one intermediate medium exchange. The assay evaluates the ability of the cells to convert resazurin into resofurin, so for the reaction we provided 100 μl fresh medium and added 20 μl viability reagent. We incubated the cells for 1 hour in a 37 °C humidified incubator before transferring 80 μl to a black 96-well plate. Fluorescence intensity was then measured using the FLUOstar OPTIMA (BMG Labtech, Germany) fluorometer. Excitation was at 560 nm and emission was at 590 nm.

### Senescence-associated β-galactosidase activity

For assessment of pH-dependent senescence-associated β-galactosidase (SA-β-gal) we applied the SA-β-gal staining kit (9860; Cell Signaling Technology, USA). We followed the manufacturer’s instructions to stain MSC in early and late passages. In parallel, SA-β-gal activity was assessed by flow cytometry as described by Debacq-Chainiaux et al. [39]. MSC were seeded at 1 × 10^3^ cells per cm^2^ in 24-well plates. After 7 days, the cells were incubated with 100 nM Bafilomycin A1 (Sigma, Germany) for 1 hour to alkalize the lysosomes. Next cells were incubated with 2 mM of the fluorogenic substrate C12FDG (Invitrogen, Germany) for 2 hours at RT. Fluorescence was acquired on a FACSCanto II (BD Biosciences, Germany) and data were analyzed using FlowJo (Tree Star Inc., USA).

### Generation of three-dimensional collagen gels and collagen contraction quantification

Three-dimensional collagen gels were prepared as published previously [40, 41] by mixing eight volumes of acidic collagen G (3 mg/ml collagen I/III in 12 mM hydrochloric acid; Biochrom, Germany) with one volume of DMEM 4.5 g/l d-glucose 10× concentrated (Biochrom, Germany). Sodium hydroxide (2 M) was then used to neutralize the mixture and one volume of culture medium containing 1 × 10^6^ MSC per ml was added into the mixture. Gels were polymerized at 37 °C in a humidified atmosphere for 1 hour after 500 μl of the collagen mixture was poured into each well of a 24-well plate. MSC within the gels were fed by adding 500 μl of the respective medium to top of the polymerized gels, which were kept for 28 days at 37 °C in a humidified atmosphere with two weekly medium changes. Next, gels were fixed in formaldehyde for 24 hours at 4 °C and contraction was quantified after photographing (Discovery V12, Germany) and measuring the circular area of each gel (ImageJ, open source). Collagen area is expressed as the percentage of the total collagen area of the collagen gels without cells.

### Extracellular matrix remodeling

ECM remodeling was assessed by performing immunohistochemistry (IHC) of the three-dimensional collagen gels containing MSC of different sources as described previously [40]. Formaldehyde-fixed gels were cut in half and with a cross-sectional view upward for paraffin embedding. The gels were sliced in 3-μm-thick slices using a rotating microtome (Leica, Germany). For IHC we used the Dako Real detection system peroxidase/DAB+, rabbit/mouse (K5001; Dako, Germany) and stained according to the manufacturer’s recommendations. Primary antibodies used were fibronectin (FN) (1:200, F3648; Sigma, Germany) and osteopontin (OPN) (1:500, sc-21742; Santa Cruz Biotechnology, Germany). Slides were dehydrated and mounted in Vitro-Clud (Langenbrinck, Germany). Images were acquired with a Cool Snap™ HQ2 digital camera (Photometrics, USA) on an Axiophot 2 microscope (Carl Zeiss, Germany).

### Flow cytometry

MSC from different sources were analyzed by flow cytometry for a wide panel of markers, as presented in Table 1. The staining procedure was performed as described previously [36, 40, 41]. A minimum of 100,000 events was acquired on FACSCanto II (BD Biosciences, Germany) and the data were analyzed using FlowJo (Tree Star Inc., USA).

### Immunofluorescence

For immunofluorescence, 1 × 10^3^ MSC per cm^2^ were seeded into 24-well plates and after 24 hours fixed with 4% paraformaldehyde (PFA) for 20 minutes at RT. Next cells were permeabilized with 0.2% Triton X-100 for 30 minutes and kept at 4 °C overnight in PBS. Primary antibody (Table 1) incubation was done overnight at 4 °C. Secondary antibodies donkey anti-rabbit (A21206; Thermo Fisher, Germany) or goat anti-mouse (A11001; Thermo Fisher, Germany) were used at 1:100 for 1-hour incubation at RT. Rhodamine-TRITC (50 μg/ml, P1951; Sigma, Germany) incubated for 40 minutes at RT was used for staining the MSC F-Actin fibers. Fluorescence was acquired with a DMI 6000B microscope (Leica, Germany).

### Telomere length analysis by quantitative fluorescence in-situ hybridization

Telomere length was assessed by quantitative fluorescence in-situ hybridization (Q-FISH) as described previously [42–44]. MSC from different sources in early and late passages were analyzed in parallel. Cells were fixed in methanol/acetic acid (3:1), cytospun, air dried, and dehydrated with ethanol before telomeres were stained with a Cy3-(C3TA2) peptide nucleic acid (PNA) probe (Panagene, South Korea). After 2 hours of incubation at RT in a humidified chamber, cells were washed with formalin-based buffer 2× for 15 minutes and DAPI was used for nuclei counterstain. Slides were mounted with Vectashield antifade mounting medium (Vector Labs, USA) and fluorescence was acquired with the high-resolution laser-scanning microscope LSM710 (Zeiss, Germany). Images were captured at 63× optical magnification with additional 1.2× zoom. A multi-tracking mode of 0.5-μm steps was used to acquire images of DAPI and Cy3 staining. Maximum projection of five single consecutive steps was done for TL quantification using Definiens software (Definiens, Germany). Nuclei and telomeres were detected based on the respective DAPI and Cy3 intensity. Due to the impossibility of calculating an age-adapted telomere length, the absolute telomere signal was used to calculate telomere loss (Δtelomere length) per passage in arbitrary units (a.u.) of fluorescence.

### Epigenetic aging signature

The aging signature (EAS) introduced by Weidner et al. [45] uses bisulfite pyrosequencing to assess DNA methylation (DNAm) levels at three CpG sites located in the genes *ITGA2B*, *ASPA*, and *PDE4C*. For EAS analysis, genomic DNA (gDNA) was first isolated using the DNA blood kit (Qiagen, Germany) and then cleaned using the clean & concentrator kit-5 (Zymo Research, USA). Five hundred nanograms of gDNA was used for further bisulfite conversion and pyrosequencing at Varionostic GmbH (Ulm, Germany). Pyrosequencing results were used for age prediction by applying the following multivariate linear regression model:

$$ \mathrm{Age}\left(\mathrm{years}\right)=38.0-26.4\alpha -23.7\beta +164.7\gamma . $$

This equation was designed to predict age with a mean absolute deviation from chronological age of less than 5 years.

### Real-time quantitative PCR

Quantification of mRNA expression for the assessed genes was performed by real-time quantitative PCR (qRT-PCR) using the 7300 Real-Time PCR System (Applied Biosystems, Germany). Total RNA was extracted using the universal RNA Purification Kit (Roboklon, Germany). Complementary DNA (cDNA) synthesis was performed using the cDNA Reverse Transcriptase Kit (Applied Biosystems, Germany). Amplification consisted of initial denaturation at 95 °C for 10 minutes, followed by 40 cycles of denaturation at 95 °C for 15 seconds and final extension at 60 °C for 1 minute. The housekeeping gene *GAPDH* was used for data normalization. Gene expression was set to one on human embryonic stem cells (total RNA, 5825; Sciencell, USA) using the 2^−ΔΔct^ method. The primers used are presented in Table 2. Seven CV-MSC donors between passages 2 and 4 were screened for pluripotency genes.

**Table 2:** Primers used for real-time quantitative PCR

	Forward sequence (5′–3′)	Reverse sequence (5′–3′)
*GAPDH*	GAAGGTGAAGGTCGGAGTCA	AATGAAGGGGTCATTGATGG
*SOX2*	GAGAGTGTTTGCAAAAGGGG	TGGGGCTCAAACTTCTTCTC
*SOX2* (2)	CCACCTACAGCATGTCCTACTCG	GGGAGGAAGAGGTAACCACAGG
*OCT4*	GCAGAAGAGGATCACCCTGG	AAAGCGGCAGATGGTCGTTT
*OCT4* (2)	CTGCACAGATATGCAAAGCAG	TGATCTGCTGCAGTGTGGGT
*NANOG*	CTTGCCTTGCTTTGAAGCAT	TTCTTGAC**C**GGGACCTTGTC
*NANOG* (2)	ACCTCAGCTACAAACAGGT	AAAGGCTGGGGTAGGTAGGT
*hTERT*	CGGAAGAGTGTCTGGAGCAA	GGATGAAGCGGAGTCTGGA
*hTERT* splicing variant	GCC TGA GCT GTA CTT TGT CAA	CGC AAA CAG CTT GTTCTC CAT GTC
ERK1/2 (MAPK1)	GCTAGATTCCAGCCAGGATACA	AGAACACCGATGTCTGAGCA
AKT1	ATGAGCGACGTGGCTATTGT	CCTCACGTTGGTCCACATCC
CSHL1	CTGTGGACAGCTCACCTAGC	AGCCTGGATAAGGGAACGGT
SMAD2	GTGGCAGGCGGGTCTAC	GCAAGCCACGCTAGGAAAAC
SMAD8	GTGGCCAACCTGTAGATGCC	CTCCCCAACTCGGTTGTTCA
β-Catenin	GGAGGAAGGTCTGAGGAGCAG	ATTGTCCACGCTGGATTTTCAA
SMAD1/5/9	CAGAGTGGCCAACCTGTAGA	TCCCCAACTCGGTTGTTCAG

*GAPDH* glyceraldehyde 3-phosphate dehydrogenase, *SOX2* sex determining region Y-box 2, *OCT4* octamer-binding transcription factor 4, *hTERT* human telomerase reverse transcriptase, *ERK* extracellular signal-regulated kinase 2, also known as mitogen-activated protein kinase 1 (*MAPK1*), *AKT* protein kinase B, *CSHL* chorionic somatomammotropin hormone like 1, *SMAD*, mothers against decapentaplegic homolog

### *hTERT* mRNA and *hTERT* splicing variant detection

To determine human telomerase reverse transcriptase (*hTERT*) mRNA levels, we performed qRT-PCR under the same conditions as before except for the use of 250 ng total RNA per reaction and the use of the following cycling conditions: 50 °C for 30 minutes, 95 °C for 15 minutes, 94 °C for 45 seconds, 60 °C for 45 seconds, 72 °C for 90 seconds for 31 cycles, and 72 °C for 10 minutes [46]. Primers are presented in Table 2. To detect *hTERT* mRNA alternative splicing, we performed qRT-PCR as before [47]. Splice variant products were amplified from 3.5 μl cDNA using primers hT2164F and hT2620R, also presented in Table 2. Cycling conditions were 94 °C for 15 minutes, 95 °C for 30 seconds (40 cycles), 64 °C for 45 seconds, 72 °C for 45 seconds, and 72 °C for 5 minutes. Primers hT2164F and hT2620R generated a 457-bp product containing the A and the B reverse transcriptase motifs, designed to detect the presence of α and β deletions. Total human embryonic stem cell RNA (5825; Sciencell, USA) was used as a positive control.

### Western blot analyses

Total protein was extracted from MSC using RIPA buffer containing protease inhibitor cocktail (Roche, Germany) and quantified using the BCA protein assay (Thermo Fisher, Germany). NuPAGE Novex 4–12% gradient Bis–Tris gels (Thermo Fisher, Germany) were used for SDS-PAGE of proteins. Proteins were transferred to nitrocellulose membranes, which were blocked using 0.5× Roti-block protein-free blocking buffer (Carl Roth, Germany) for 1 hour at room temperature. Primary antibody (Table 1) incubation was done overnight at 4 °C. Secondary HRP-linked anti-rabbit/mouse/goat IgG (1:1000; Dako, Germany) with enhanced chemiluminescence (Thermo Scientific, Germany) was used for detection. Loading controls were either GAPDH (1:1000, sc-32233; Santa Cruz Biotechnology, USA) or β-Actin (1:5000, ab8227; Abcam, USA). Whole human embryonic stem cell lysate (ab27198; Abcam) was used as a positive control. Hela, NIH3T3, and Jurkat lysates were used as further controls.

### Statistical analysis

Results express the mean ± SD. Data were obtained from at least three independent donors unless stated otherwise. Statistics as well as graphical representations were performed using GraphPad Prism™ 5.0 (GraphPad Software Inc., USA). Statistical significance of data results from one-way ANOVA followed by Tukey’s post-hoc test (analysis of three or more groups). Differences were considered significant when *p* < 0.05.

## Results

### CV-MSC are highly proliferative and do not enter senescence during long-term in-vitro culture

#### Morphology and multilineage potential

**Fig. 2:**
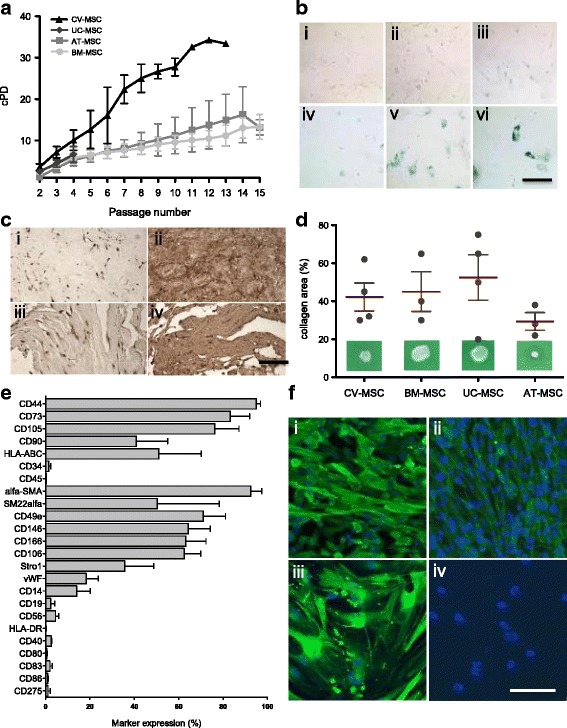
**a** Cumulative population-doubling (cPD) levels versus passage number for the four different sources of MSC. Black represents CV-MSC (*n* = 7), dark gray UC-MSC (*n* = 4), medium gray AT-MSC (*n* = 5), and light gray BM-MSC (*n* = 6). **b** IHC-based senescence-associated β-galactosidase (SA-β-gal) staining of CV-MSC in early (i, passage 4) and late (ii, passage 9) passages, AT-MSC in passage 6 (iii), BM-MSC in passage 6 (iv), and UC-MSC in passage 2 (v) and passage 4 (vi). Scale = 200 μm. **c** IHC of CV-MSC (i, ii) and BM-MSC (iii, iv) stained for osteopontin (i, iii) and fibronectin (ii, iv). Scale = 1 mm. **d** Collagen area (%) after collagen contraction assay for CV-MSC (*n* = 4), BM-MSC (*n* = 3), UC-MSC (*n* = 4), and AT-MSC (*n* = 3). Cells in passage 3 were used. Results expressed as mean ± SD, percentage of the total collagen area of the collagen gels without cells. **e** Surface marker expression of CV-MSC in early passages (*n* = 5). Results expressed as mean ± SD (%). **f** Representative immunofluorescence of early passaged CV-MSC (i, iii) and BM-MSC (iii, iv) stained for SM22α (i, iii) and α-SMA (ii, iv). Scale = 50 μm. AT adipose tissue, BM bone marrow, CV chorionic villi, MSC mesenchymal stromal cells, UC umbilical cord

We isolated a MSC population of fetal origin from CV tissue of the placenta using collagenase digestion (Fig. 1a i–v). The isolated CV-MSC were assessed for the absence of maternal cell contaminations at passages 3–4 (early) and 8–10 (late) using FISH X/Y chromosome analysis. The male newborn karyotype was confirmed for every CV-MSC donor used in the study by the presence of an X/Y chromosome pair (Fig. 1a iv–v). The amount of cells obtained after digestion of a maximized weight of 200 g (wet weight) CV tissue per placenta resulted in approximately 5.40 × 10^6^ ± 6.00 × 10^5^ cells. These cells were expanded to 8.05 × 10^7^ ± 1.2 × 10^6^ cells for the first passage and 1.50 × 10^8^ ± 1.02 × 10^7^ cells by the second passage. In the second passage, CV-MSC reached a cumulative cell number corresponding to the highest PD number (3.6 ± 0.7 PD). BM-MSC reached 2.6 ± 0.6 PD, AT-MSC reached 1.4 ± 1.1 PD, and UC-MSC reached 2.5 ± 0.3 PD (Fig. 2a).

The absence of ECM, endothelial cells, hematopoietic progenitors, or blood cells in the isolated CV-MSC was verified microscopically after the first passage (Fig. 1b). CV-MSC morphology did not vary with increasing passages (Fig. 1b i, iii, iv). However, the morphology differs from the typical spindle-shaped morphology of BM-MSC (Fig. 1b v, vi), UC-MSC, and AT-MSC (Additional file 1: Figure S1B i, ii), probably relating to a more heterogeneous cell population (Fig. 1b ii).

Multilineage differentiation potential of CV-MSC was confirmed as shown in Fig. 1c i–iii and no significant differences in the potential for osteogenic, chondrogenic, and adipogenic differentiation were observed between CV-MSC, BM-MSC, UC-MSC, and AT-MSC. The latter cell source showed increased adipose differentiation potential (Additional file 1: Figure S1B i–ix).

#### Long-term growth, proliferation potential, and senescence

Long-term cultures shown in Fig. 2a were monitored during 15 passages. While CV-MSC reached 8 ± 4 passages within 138 ± 64 days on average, corresponding to an average of 25 ± 3 cPD (Fig. 2a; *n* = 7; *p* < 0.05), BM-MSC reached on average 12 ± 6 passages within 329 ± 144 days, corresponding to an average of 10 ± 2 cPD (*n* = 6; *p* < 0.05). AT-MSC donors reached passage 12 approximately within 177 days, corresponding to an average number of 13 ± 5 cPD (*n* = 5), and could be expanded up to 32 passages, corresponding to an average period of 383 ± 10 days in culture and more than 30 cPD (not shown) until the cultures were intentionally stopped. UC-MSC could not be viably maintained for more than four passages (average 4 ± 2 passages; *n* = 4), corresponding to an average period of 78 ± 5 days in culture and an average of 7 ± 1 cPD. When we compare all sources after only four passages, CV-MSC, BM-MSC, UC-MSC, and AT-MSC reached 10 ± 2 cPD, 6 ± 1 cPD, 7 ± 1 cPD, and 5 ± 1 cPD, respectively (Fig. 2a). After 12 passages the differences are dramatic, with CV-MSC having reached 34 ± 3 cPD, BM-MSC reaching 11 ± 2 cPD, and AT-MSC 13 ± 5 cPD (Fig. 2a). The higher proliferation ability of CV-MSC compared to all other cell sources was substantiated by a 7-day in-vitro cell viability assay showing that CV-MSC grow 2.85 ± 0.55 times faster than BM-MSC (*p* = 0.02) (Additional file 2: Figure S2A).

We next looked for β-gal-positive senescent cells in early and middle-to-late passages of CV-MSC and observed only marginal cell senescence from passage 3 until passage 9 (β-gal-positive cells = 7.7 ± 1.5% at passage 9; Fig. 2b, Additional file 2: Figure S2B). BM-MSC and AT-MSC showed the first signs of senescence in passage 6 (Fig. 2b), while UC-MSC reached senescence much earlier during culture, starting at passage 2 (β-gal-positive cells = 17.7 ± 2.4% at passage 4; Fig. 2b, Additional file 2: Figure S2B).

### CV-MSC are heterogeneous populations demonstrating a typical MSC phenotype and remodeling potential

#### Matrix remodeling and immunophenotype

Next we assessed ECM remodeling ability by culturing the cells for 28 days using our collagen-based system described previously [40, 41]. CV-MSC showed equivalent ability to BM-MSC to spontaneously produce OPN and FN, two main ECM remodeling proteins, in long-term in-vitro cultures (Fig. 2c). The degree of ECM production of MSC from different sources in vitro was next quantified using the collagen contraction assay (Fig. 2d; Additional file 2: Figure S2C). Evaluation of collagen areas after contraction confirmed that CV-MSC (42.5 ± 7.4%, *n* = 4) and BM-MSC (45.0 ± 10.4%, *n* = 3) have similar remodeling potential. Weaker contraction was obtained by UC-MSC (52.50 ± 12.0%, *n* = 4) and stronger contraction by AT-MSC (29.3 ± 9.6%, *n* = 3), compared to CV-MSC.

Immunophenotypic characterizations of CV-MSC in past studies were inconsistent. We applied a wide panel of markers associated not only with MSC of BM origin but also human AT, endothelial cells, smooth muscle cells (SMC), hematopoietic cells, and pericytes to fully characterize MSC from different origins. Our flow cytometry data (Fig. 2e; Table 1) show that CV-MSC: express phenotypic markers that fit the panel defined by the International Society for Cellular Therapy (ISCT) to define human multipotent mesenchymal stromal/stem cells [48] expressing CD44, CD73, CD105, CD90, and HLA-ABC and lacking expression of CD45, CD34, CD19, and HLA-DR surface molecules; possess the nonimmunogenic character of MSC by lacking expression of the immune markers CD14, CD56, CD80, CD83, CD86, CD40, and CD275 (B7-H2) [49]; show a vascular expression pattern by strongly expressing STRO-1 and CD146, which are markers for vascular niches [50]; and are prone to SMC lineage commitment by expressing high levels of α-SMA (92.5 ± 5.5%) and CD146 (64.3 ± 9.4%). SM22α was positive (approximately 50% expression) for all MSC sources (Table 3). Additionally, early myogenic markers were corroborated by immunofluorescence, which revealed positive α-SMA expression—associated with actin-positive stress fibers—and concurrent expression of SM22α in early-passage CV-MSC (Fig. 2f i, iii), consistent with increased CV-MSC potential for myogenic lineage commitment compared to BM-MSC. The late myogenic markers SM-MHC and Calponin were absent both in CV-MSC and BM-MSC (data not shown). In contrast, BM-MSC expressed only marginal STRO-1 and CD146 expression, and showed low expression of SMC markers (α-SMA^+^ 18.4 ± 2.8%; Fig. 2f ii, iv; Table 3).

**Table 3:** Immunophenotypic characterization of mesenchymal stromal cells

	CV-MSC (%)	BM-MSC (%)	UC-MSC (%)	AT-MSC (%)
CD44	94.9	97.4	93.3	93.9
CD73	83.4	74.2	82.2	96.4
CD105	76.3	95.7	66.3	94.0
CD90	40.8	81.0	85.8	96.9
HLA-ABC	51.1	81.0	81.9	81.0
CD34	1.4	0.5	1.5	2.5
CD45	0.3	0.2	3.4	0.1
α-SMA	92.5	18.4	9.8	27.5
SM22α	50.5	48.4	–	45.7
CD49a	71.2	91.2	52.7	–
CD146	64.3	18.8	52.1	63.1
CD166	63.2	73.1	53.6	85.2
CD106	62.5	68.0	61.2	4.3
Stro-1	44.0	9.8	2.6	2.3
vWF	18.4	12.5	6.3	6.2
CD14	14.1	2.0	1.1	0.1
CD19	2.4	0.5	0.3	–
CD56	4.5	3.5	2.8	2.0
HLA-DR	0.3	0.2	0.2	0.2
CD40	2.6	2.8	1.6	–
CD80	0.6	0.4	0.7	–
CD83	2.0	3.0	2.6	–
CD86	0.9	0.3	0.2	–
CD275	1.1	0.2	0.1	–
CD16	–	–	–	5.6
CD31	–	–	–	0.1
CD49d	–	–	–	42.2
CD13	–	–	–	75.2
CD29	–	–	–	98.2

Results expressed as mean percentage of marker expression. CV-MSC of passages 3–5 (*n* = 5), BM-MSC of passages 2–3 (*n* = 4), UC-MSC of passage 2 (*n* = 4), AT-MSC of passages 2–4 (*n* = 8)

*AT* adipose tissue, *BM* bone marrow, *CV* chorionic villi, *MSC* mesenchymal stromal cells, *UC* umbilical cord

As in other studies, the possibility of the presence of a subpopulation of pericyte-like cells or pericytes within the isolated and further cultured CV-MSC is strong in our study. This is because cultured CV-MSC have shown increased combined expression of CD146 and Stro-1 compared to the other cell sources (Table 3). However, the combined expressed markers alone do not prove the presence of pericytes.

CD49e (71.20 ± 11.7%), CD166 (62.30 ± 8.0%), and CD106 (62.5 ± 5.0%) were strongly expressed in CV-MSC, which is in line with previous studies [2, 4, 18, 49–51]. No difference was found among the expression of these three markers on cells from the different sources.

### CV-MSC undergo reduced telomere loss delaying aging with increasing passages

#### Telomere length and methylation status

**Fig. 3:**
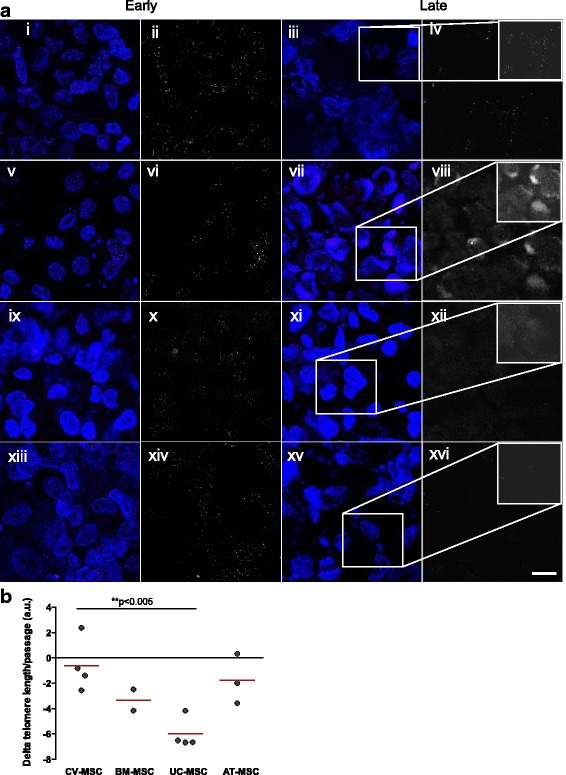
**a** Confocal microscopy of early and late-passage CV-MSC (i–iv), BM-MSC (v–viii), AT-MSC (ix–xii), and UC-MSC (xiii–xvi) analyzed by Q-FISH. Telomeres were stained with Tel-Cy3 peptide nucleic acid probe and chromatin was stained with DAPI (overlaid image on left). Telomere signal alone (black and white image on right) shown to improve visual comparability. Shown side by side are on the left MSC in passage 3 (i, ii, v, vi, ix, x) and on the right MSC in passage 9 (iii, iv, vii, viii, xi, xii), except for UC-MSC that were on the left in passage 1 (xiii, xiv) and on the right in passage 3 (xv, xvi). Scale = 10 μm. **b** Telomere loss (Δtel) per passage (in arbitrary units (a.u.)) according to Q-FISH. CV-MSC (*n* = 4), BM-MSC (*n* = 2), UC-MSC (*n* = 4), AT-MSC (*n* = 3) (**p < 0.005). AT adipose tissue, BM bone marrow, CV chorionic villi, MSC mesenchymal stromal cells, UC umbilical cord

In order to understand whether the increased proliferative potential observed in CV-MSC is being recapitulated by altered telomere maintenance, we sequentially analyzed telomere length. The most dramatic telomere loss was observed in UC-MSC (–6.00 ± 0.61 a.u.; *n* = 4; two passages analyzed) (Fig. 3a, b). This was particularly dramatic, as UC-MSC could not be maintained in culture for more than four passages. As the cells aged in culture, CV-MSC suffered the least pronounced telomere loss per passage (–0.59 ± 1.055 a.u.; *n* = 4; five passages analyzed) (Fig. 3a, b). In fact, multiple intense fluorescent telomeric signals within the CV-MSC could be observed by the naked eye in early and late passages under LSCM (Fig. 3a i–iv), in contrast to all other MSC sources (Fig. 3a v–xvi). AT-MSC suffered the second least pronounced telomere loss (–1.74 ± 1.13 a.u.; n = 3; six passages analyzed), while BM-MSC suffered the third least pronounced telomere loss (–3.32 ± 0.84 a.u.; n = 2; five passages analyzed).

Next we applied the EAS as an attempt to understand whether or not the predicted aging at the telomere level will match predicted aging at the methylation level. Results showed that all cell sources were predicted to be of a far different age than their chronological age, with differences ranging from ca. 16 to 38 years either older or younger (Additional file 3: Figure S3A–C). Age predictions according to DNAm changes seem to be particularly inaccurate in the case of CV-MSC both in early and in late passages (Additional file 3: Figure S3B, C, respectively). Interestingly the predictions seem to corroborate—at least in part—the predictions based on telomere data. Predicted age in CV-MSC rapidly decreased with progressive in-vitro passaging (Additional file 3: Figure S3A), suggesting a delayed aging phenomenon already observed at the telomere level. In opposition, predicted age of all other MSC types stabilized or increased with increasing passages (Additional file 3: Figure S3A). This is in line with the establishment of an aging phenotype characterized by telomere shortening, decreased proliferation, and senescence onset.

### CV-MSC express no full hTERT or shorter splicing variants

#### hTERT

**Fig. 4:**
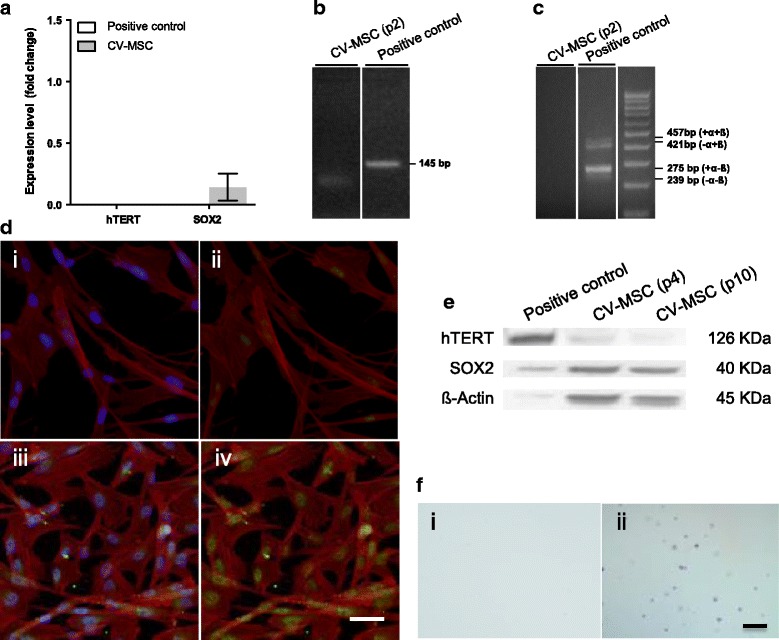
**a** Relative mRNA *hTERT* and *SOX2* expression in passage 2 of CV-MSC. Data calibrated to embryonic stem cells (hESC) control, expression of which is considered one for both genes. Normalization to the housekeeping gene *GAPDH*. Results expressed as mean ± SD. **b** RT-PCR product for *hTERT* (145 bp) in CV-MSC passage 2 and hESC positive control run on 2% agarose gel. **c** *hTERT* splicing variant RT-PCR products in hCMSC passage 2 and hESC control run on 2% agarose gel. hTERT was 457 bp in full length (+α + β) and 421 bp (–α + β), 275 bp (+α–β), and 239 bp (–α–β) in the variants. **d** Immunofluorescence CV-MSC in passage 4 stained for hTERT (i, ii) and SOX2 (iii, iv). Primary antibodies labeled with Alexa Fluor 488 (i–iv). Cells nuclei counterstained with DAPI (i, iii) and F-actin fibers with Rodhamine-TRITC (i–iv). Scale = 10 μm. **e** Western blot analysis to detect hTERT and SOX2 proteins in CV-MSC in passage (p) 4 and 10. hESC as positive control included. **f** Representative images of passage 4 hCMSC (i) after subjecting to soft agar assay. A tumorigenic cell line, LN319, included as positive control, developing tumoroids identifiable after 4 weeks (ii). Scale = 50 μm. CV chorionic villi, MSC mesenchymal stromal cells, *hTERT* human telomerase reverse transcriptase, *SOX2* sex determining region Y-box 2

To understand whether hTERT is the reason for the good maintenance of telomere length in late-passage CV-MSC we investigated hTERT mRNA expression. No detectable hTERT mRNA was observed in CV-MSC at passage 2 or later (Fig. 4a, b). No shorter hTERT splicing variants were detected either (Fig. 4c). At the protein level we found no indications for the production of hTERT protein in passage 4 CV-MSC using either a full-length peptide hTERT antibody (western blot analysis) or a peptide selected form the center region of hTERT (immunofluorescence) (Figure 4d i, ii, e).

### CV-MSC express SOX2 but no other pluripotency markers

#### Pluripotency markers

Pluripotency of CV-MSC has been claimed by different groups [7–9]. In order to assess pluripotency of CV-MSC we first analyzed the expression of three genes, *NANOG*, *OCT4* variant 1 (POU5F1), and *SOX2*, associated with pluripotency and typically present in ESC. Analysis of mRNA expression of CV-MSC in passage 2 revealed a weak detectable expression of *SOX2* (0.14 ± 0.11-fold change; *n* = 4) but no expression of *OCT4* or *NANOG* (data not shown). On the protein level, we observed that CV-MSC maintained SOX2 protein expression throughout passaging (at least until passage 10; Fig. 4e), but no OCT4 isoform 1 or NANOG protein was detected (Additional file 4: Figure S4B).

Last we applied the soft agar assay in order to assess tumorigenicity in vitro, and observed no evidence for this as CV-MSC yield no tumoroids after 4 weeks in soft agar cultures (Fig. 4f).

To uncover any clue on whether there is evidence of pluripotency in CV-MSC, we looked into several signaling pathways typically involved in ESC pluripotency and self-renewal. While mRNA and protein profiles of CV-MSC were investigated extensively, no difference was observed in *Erk1/2*, *Akt1*, *Smad2*, *Smad9*, *β-Catenin*, *Smad1/5/9*, and *CSHL* gene expression between CV-MSC and BM-MSC (Additional file 4: Figure S4C). Western blot analysis confirmed gene expression results (Additional file 4: Figure S4D, E).

## Discussion

We have successfully isolated a highly proliferative MSC population from CV of postnatal placenta while optimizing our protocols to maximize cell yield from extraction (approximately 2.7 × 10^7^ cells/kg wet weight of CV) and after a few weeks of in-vitro culture (approximately 7.5 × 10^9^ cells/kg wet weight of CV, after 3.6 ± 0.3 cPD). We kept CV-MSC in culture for an extended period (138 ± 64 days, corresponding to 25 ± 3 cPD) and observed delayed onset of the earlier stages of cell senescence compared to BM-MSC. In line with Barlow et al. [23], we observed that CV-MSC proliferate faster and show greater long-term growth ability than BM-MSC.

Collagenase digestion was used for cell isolation in our study so associated costs must not be overlooked as they certainly rise above those of the concurrent explant method when digesting maximized volumes of placenta tissue is a priority. However, collagenase digestion has the advantage of allowing for higher final cell yields in a short period of time. Adapted or alternative methods for isolation of placental MSC of fetal origin have been proposed [52, 53]. The preferred isolation method and in-vitro culture conditions must be assessed according to the specific requirements of each given stem cell application.

We have extensively characterized the CV-MSC cultured fraction in early and late passages. It is obvious that CV-MSC fit all MSC defining criteria [48], as reported previously [3, 50, 51, 54], including being able to differentiate into osteoblasts, adipocytes, and chondrocytes cultured in vitro in standard differentiation cocktails. We also observed that CV-MSC present a surface marker profile consistent with increased potential for proliferation (high CD44, CD73, and CD166 combined expression), vascular localization (high STRO-1, CD146, and CD106 combined expression), and predisposition for myogenic commitment (high α-SMA, SM22α, and CD146 combined expression), unlike BM-MSC. It has been introduced that combined CD146 expression and a high α-SMA expression is associated with SMC commitment in BM-MSC [55]. Myogenic expression of CV-MSC, however, remains another topic of debate, as some studies report residual expression of α-SMA in MSC isolated from placenta tissues [56, 57] while others, such as Castrechini et al. [50], report high expression but just in CV-MSC isolated from first-trimester pregnancies.

Numerous studies report that CV-MSC isolated by collagenase digestion are prone to contamination by maternal cells, which rapidly and completely overgrow CV-MSC within one or two passages [23, 58, 59]. To address this issue we have excluded by FISH X/Y chromosome analysis the presence of maternal contamination in late-passage CV-MSC.

An often overlooked contributor to an even more complete phenotypic characterization is trophoblastic contamination in the cultures. Trophoblastic cells are the cells forming the outer layer of the blastocyst that provide nutrients to the embryo. This layer later develops into a large part of the placenta and so trophoblast-derived cells make up the majority of the chorion in the developed placenta. To determine cytotrophoblastic cells (eventually the most frequent trophoblastic cell type) in vitro, markers such as human chorionic gonadotropin, pan-cytokeratin, epidermal growth factor receptor HER2, and E-Cadherin could be investigated. Despite the controversy, most indications for trophoblastic contaminations are unlikely [20, 50].

In terms of ECM remodeling potential, CV-MSC showed the ability to produce matrix proteins (namely FN and OPN) and contract a collagen matrix in vitro similar to BM-MSC, confirming CV-MSC suitability for major stem cell-based applications.

To understand whether the observed increased potential of CV-MSC to proliferate correlated with increased telomere length, we analyzed the telomeres, repeated TTAGGG sequences at the ends of chromosomes that protect them from deterioration or fusion. Telomeres undergo progressive shortening with each cell division. Progressive telomere shortening is therefore one of the molecular mechanisms underlying aging, as critically short telomeres trigger cellular senescence and loss of cell viability [60–62]. Thus, telomere length is known to decline during in-vitro and in-vivo aging. Our results showed that CV-MSC undergo minimal telomere loss as they age in vitro compared to all other MSC sources. In contrast, UC-MSC underwent dramatic telomere loss within the very few passages we were able to keep them in vitro. At the same time we observed that UC-MSC become quickly senescent (i.e., in early passages) in β-galactosidase senescence assays. Interestingly, CV-MSC escaped in-vitro senescence—showing reduced β-galactosidase-positive cell rates—for more passages than all the other cells types due to increased potential to maintain telomere length while proliferating. The increased potential of CV-MSC to maintain telomere length over the other tissue sources reflects not only different rates of telomere loss within those tissues, but also different telomere loss rates during in-vitro passaging, potentially due to oxidative stress. Given that both CV-MSC and UC-MSC originate from postnatal tissues with virtually equivalent chronological age but dramatically different telomere length maintenance potentials, it is not clear to us why telomere erosion during in-vitro culture was so dramatic in UC-MSC. It can be related to donor variability, differential telomerase activity expression, or alternative mechanisms of telomere maintenance, as discussed later.

DNAm is known to change during aging. However, some CpG sites show almost linear changes during aging and so can be used for age prediction. Weidner et al. [45] have established an EAS to predict aging with higher precision than telomere length alone. EAS revealed predicted age in CV-MSC decreased rapidly with progressive passaging, confirming delayed aging phenomena. It is worth mentioning that our results were apparently limited by the fact that the signature was designed for age estimations from blood samples and does not seem to suit cultured cells. This leads us to a discussion based on the analysis of linear regression fits and not on absolute predictions (which fall out of the acceptable chronological age range).

*hTERT* is a catalytic subunit of the enzyme telomerase, which together with the telomerase RNA component (*hTERC*) comprises the most important unit of the telomerase complex. *hTERC *acts as a template for the addition of telomere units by *hTERT*. *hTERT* is expressed during early development but is absent in most somatic cells, with the exception of proliferating cells and renewal tissues [63]. In highly proliferative cells of the germline, in ESC, and in the majority of cancer cells, telomerase (by adding telomeric repeats onto the chromosome ends) prevents the replication-dependent loss of telomeres and cellular senescence [61]. The causal relationship between expression of telomerase, maintenance of telomere length, and elongated life span of the human cell has been established. We have confirmed the absence of *hTERT *mRNA in CV-MSC. *hTERT* gene expression typically corresponds to telomerase activity in many multicellular organisms. This can be untrue in some cases. Izadpanag et al., Yanada et al., Zimmermann et al., and Hiyama and Hiyama [64–67] reported that low levels of telomerase activity were found in MSC. Contradictory studies report no telomerase activity in MSC [68]. Therefore, a mechanism other than or in addition to telomerase—such as alternative lengthening of telomeres (ALT)—might play an important role in CV-MSC telomere maintenance. There are, for instance, hints from work done on subtelomeric DNA hypomethylation facilitating telomere elongation in mammalian cells suggesting that epigenetic modifications of chromatin might occur in MSC [69]. Work done in whole chorion tissues indicates a downregulation of telomerase activity over the gestation, also supporting the idea of a decline of primitive stem cell features with aging [70]. In order to clarify the origin of our telomere observations, investigating telomerase activity levels and ALT mechanisms in CV-MSC would thus be an interesting outlook.

Previous studies using equivalent methods for isolation of CV-MSC [7–9] report the presence of panels of pluripotent markers such as *NANOG*, *OCT4*, and *SOX2* in those cells. Studies typically focus on gene expression level observations only and often PCR primer sequences or expression data are omitted. Without further concerns, some conclude that CV-MSC retain characteristics of pluripotent stem cells.

We have designed qRT-PCR primers binding to the DNA region encoding for the highly conserved AFMVW helix inside the HMG domain of the SOX2 protein. Additionally, we used *SOX2* primers to bind the DNA region encoding for the C-terminal domain of the SOX2 region, equivalent to what was done in other studies [71, 72]. Our data show that CV-MSC express SOX2 on both the gene and protein levels, an indication of improved neurogenic potential in the light of current knowledge. *SOX2*, a pluripotency marker, is also known to regulate FGF4 expression, which in turn promotes neural stem cell proliferation and differentiation in the postnatal brain [73]. The improved neurogenic potential of CV-MSC compared to BM-MSC has in fact been demonstrated [18, 20].

We did not detect the presence of *OCT4* variant 1 or *NANOG* in CV-MSC. Our data are partially supported by previous work from Jones et al. [72], who compared first-trimester to term fetal placental chorionic stem cells. They observed no detectable *OCT4A *variant 1 in the term fetal cells at the transcript level using primer pairs binding only to a larger DNA fragment within the same region as ours. Based on the absolute expression of *NANOG* reported in that work, and given the fact that one of the two primer pairs we used was equivalent to theirs, we consider the possibility of marginal but nondetectable expression of *NANOG* in our cells due to donor variability.

We nonetheless commit to exclude the possibility of pluripotency in CV-MSC given the corroborated absence of *OCT4A* variant 1. *OCT4* and *SOX2* were identified as the fundamental transcriptor factors underpinning naïve pluripotency [74], although the critical role of SOX2 might be to activate *OCT4 *[75, 76]. *NANOG* becomes part of the *OCT4/SOS2/NANOG* (OSN) triumvirate as its presence is crucial for the acquisition but not the maintenance of naive pluripotency [77, 78].

One other indisputable feature of pluripotent stem cells is the formation of teratomas in vivo. We applied the equivalent in-vitro assay, designed to assess the tumorigenic potential of cells in culture—the soft agar assay—and found no evidence for malignant transformation of CV-MSC, as suggested previously [16, 79].

In human ESC the predominant signaling pathways involved in pluripotency and self-renewal [80] are TGF-β (signaling through SMAD2/3/4, activating the MAPK and AKT pathways) and the noncanonical WNT pathway (β-CATENIN signaling). Pluripotency signaling through these pathways relies predominantly upon the key transcription factors *OCT4*, *SOX2*, and *NANOG*. When NANOG is inhibited, differentiation takes place via the BMP pathway (Smad1/5/9 signaling) and NOTCH intracellular domain (NICD, CSHL1). After investigating multiple pathways we found no evidence of any pathways being differentially activated/deactivated leading to pluripotency of CV-MSC.

Placental-derived MSC have been reported to be capable of neural, retinal cell, pancreatic progenitor cell, and hepatic cell differentiation [8, 81], an indication for greater plasticity. We have no evidence, however, to support the notion that a putative pluripotent stem cell population is present within CV-MSC or that CV-MSC are less differentiated than BM-MSC.

## Conclusions

In our comprehensive characterization study of CV-MSC, we show that CV-MSC hold great promise for tissue engineering applications. CV-MSC are nonimmunogenic, have multilineage differentiation potential, hold increased proliferation ability, and display a retarding aging phenotype. Our data suggest that the exceptional proliferation of CV-MSC ability might be linked with telomere length control mechanisms.

## Additional files

Additional file 1: Figure S1. **A** Bright-field microscopy images of cultured UC-MSC in passage 3 (i) and AT-MSC in passage 24 (ii). Scale = 500 μm. **B** Visualization of calcium deposits after Alizarin Red stain (i–iii, scale = 500 μm), proteogyclans after Toluin Blue stain (iv–vi, scale = 1 mm), and lipid droplets after Oil Red O stain (vii–ix, scale = 100 μm) of differentiated AT-MSC (i, iv, vii), BM-MSC (ii, v, viii), and UC-MSC (iii, vi, ix) all in passage 3. (JPG 86 kb)

Additional file 2: Figure S2. **A** Viability of hMSC of all sources during a 7-day follow-up period during early passages (passages 3–5) in culture (results expressed as arbitrary units of normalized fluorescence). Black depicts CV-MSC (*n* = 3), dark gray UC-MSC (*n* = 3), medium gray AT-MSC (*n* = 3), and light gray BM-MSC (*n* = 3). **B** Histograms for CV-MSC in passage 9 (*n* = 3) and UC-MSC in passage 4 (*n* = 3) stained for β-galactosidase assessed by flow cytometry. **C** Visualization of collagen contraction potential by CV-MSC (i), BM-MSC (ii), UC-MSC (iii), and AT-MSC (iv). All donors shown. Scale = 1 cm. **D** Immunofluorescence of early passaged BM-MSC (i, iv), UC-MSC (ii–v), and AT-MSC (iii, vi) stained for SM22α (i–iii) and α-SMA (iv–vi). Scale = 50 μm. All conditions *n* ≥ 3. (JPG 63 kb)

Additional file 3: Figure S3. **A** Difference between predicted and chronological MSC donor age (years) after EAS: CV-MSC 37.75 ± 5.43 years (*n* = 4), BM-MSC –16.00 ± 10.06 years (*n* = 4), UC-MSC 25.50 ± 1.84 years (*n* = 4), AT-MSC 17.00 ± 5.00 (*n* = 2), from passage 2 to passage 5 (**p < 0.005). **B** Difference between predicted and chronological MSC donor age (years) after EAS: CV-MSC 29.25 ± 4.46 years (*n* = 4), BM-MSC –26.40 ± 10.52 years (*n* = 5), AT-MSC 32.80 ± 9.65 (*n* = 5), from passage 6 to passage 15 (**p < 0.005). It was not possible to keep UC-MSC until late passages. **C** Predicted age (years) versus passage number EAS: one representative donor shown for CV-MSC (black), UC-MSC (dark gray), AT-MSC (medium gray), and BM-MSC (light gray). (JPG 33 kb)

Additional file 4: Figure S4. **A** Immunofluorescence CV-MSC in passage 5 stained for Oct3/4 (i) and Nanog (ii). Alexa Fluor-488 (Green) was used to label the primary antibodies (i–iv). Rhodamine-TRITC was used for F-Actin fiber labeling (i–iv) and DAPI for nuclei counterstain (i, iii). Scale = 10 μm. **B** Western blot analysis for detection of Oct3/4 and Nanog proteins in passage 4 and passage 10 CV-MSC. hESC are positive control. **C** Relative *ERK1/2*, *AKT1*, *CSHL1*, *SMAD2*, *SMAD9*, *β-CATENIN*, and *SMAD1/5/9* gene expression in CV-MSC in passage 4 and BM-MSC in passage 3. Data calibrated to positive control, expression of which is considered one for both genes. Housekeeping gene *GAPDH* used for normalization. **D, E** Western blot analysis to detect Erk1/2, pErk1/2, β-Catenin, Smad2/3, pSmad2/3, NICD, Akt1/2, pAkt, Smad1/5/9, and pSmad1/5 proteins in CV-MSC and BM-MSC. hESC, Hela, NIH3T3, and Jurkat controls included. (JPG 71 kb)

## Abbreviations

AKT: Protein kinase B
ALT: Alternative lengthening of telomeres
AT: Adipose tissue
BCA: Bicinchoninic acid
bFGF: Basic fibroblast growth factor
BM: Bone marrow
cDNA: Complementary deoxyribonucleic acid
CSHL: Chorionic somatomammotropin hormone Like 1
CV: Chorionic villi
DAPI: 4′,6-Diamidino-2-phenylindole
DMEM: Dulbecco's modified Eagle's medium
DNAm: deoxyribonucleic acid methylation
EAS: Epigenetic signature
EDTA: Ethylenediaminetetraacetic acid
ERK: Extracellular signal-regulated kinase 2 (also known as MAPK1)
ESC: Embryonic stem cells
F12: Nutrient mixture F-12
FISH: Fluorescent in-situ hybridization
FN: Fibronectin
GAPDH: Glyceraldehyde 3-phosphate dehydrogenase
gDNA: Genomic deoxyribonucleic acid
HER2: Epidermal growth factor receptor
HLA-ABC: Human leukocyte antigen, major histocompatibility complex, class I, A, B, C
HRP: Horseradish peroxidase
hTERC: Human telomerase RNA component
hTERT: Human telomerase reverse transcriptase
IHC: Immunohistochemistry
ISCT: International Society for Cellular Therapy
MAPK1: Mitogen-activated protein kinase 1 (also known as ERK)
mRNA: Messenger ribonucleic acid
MSC: Mesenchymal stromal cells
NICD: Notch intracellular domain
OCT4: Octamer-binding transcription factor 4
OPN: Osteopontin
PBS: Phosphate-buffered saline
PD: Population doubling
PFA: Paraformaldehyde
PNA: Peptide nucleic acid
Q-FISH: Quantitative fluorescent in-situ hybridization
qRT-PCR: Quantitative real-time polymerase chain reaction
RIPA: Radioimmunoprecipitation assay
RNA: Ribonucleic acid
RT: Room temperature
SA-β-gal: Senescence-associated β-galactosidade
SM22α: Smooth muscle protein 22 alfa (transgelin)
SMAD: Mothers against decapentaplegic homolog
SMC: Smooth muscle cells
SM-MHC: Smooth muscle myosin heavy chain
SOX2: sex determining region Y-box 2 (also known as SRY)
TGF: Transforming growth factor
UC: Umbilical cord
α-SMA: Alpha smooth muscle actin
