# Supplementary figures and images for: Comprehensive characterization of chorionic villi-derived mesenchymal stromal cells from human placenta
Source: Stem Cell Res Ther. 2018 Feb 5;9:28. doi: 10.1186/s13287-017-0757-1 (PMC5800083; doi:10.1186/s13287-017-0757-1)

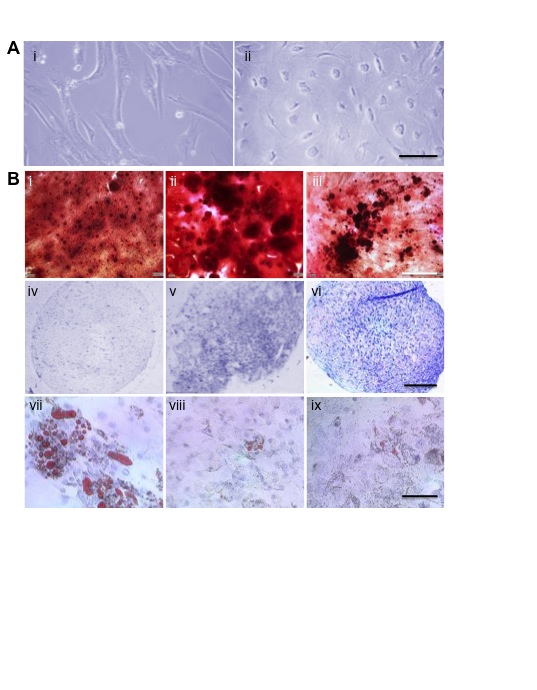

Supplement: Supplementary file 1 — A Bright-field microscopy images of cultured UC-MSC in passage 3 (i) and AT-MSC in passage 24 (ii). Scale = 500 μm. B Visualization of calcium deposits after Alizarin Red stain (i–iii, scale = 500 μm), proteogyclans after Toluin Blue stain (iv–vi, scale = 1 mm), and lipid droplets after Oil Red O stain (vii–ix, scale = 100 μm) of differentiated AT-MSC (i, iv, vii), BM-MSC (ii, v, viii), and UC-MSC (iii, vi, ix) all in passage 3. (JPG 86 kb) [file 13287_2017_757_MOESM1_ESM.jpg]

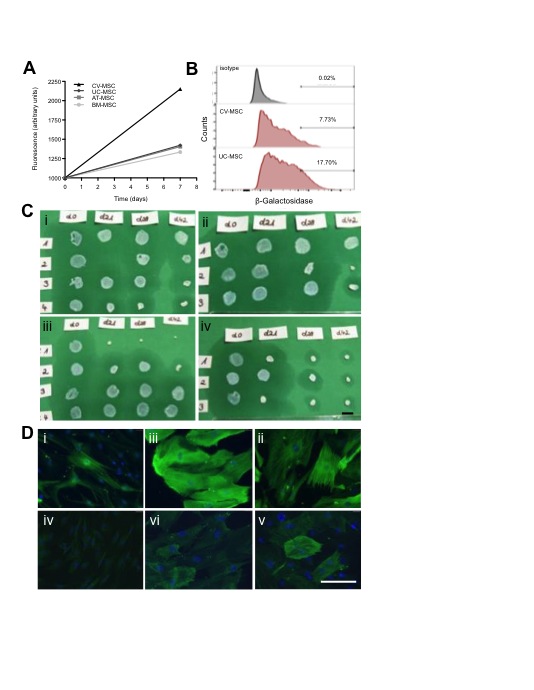

Supplement: Supplementary file 2 — A Viability of hMSC of all sources during a 7-day follow-up period during early passages (passages 3–5) in culture (results expressed as arbitrary units of normalized fluorescence). Black depicts CV-MSC (n = 3), dark gray UC-MSC (n = 3), medium gray AT-MSC (n = 3), and light gray BM-MSC (n = 3). B Histograms for CV-MSC in passage 9 (n = 3) and UC-MSC in passage 4 (n = 3) stained for β-galactosidase assessed by flow cytometry. C Visualization of collagen contraction potential by CV-MSC (i), BM-MSC (ii), UC-MSC (iii), and AT-MSC (iv). All donors shown. Scale = 1 cm. D Immunofluorescence of early passaged BM-MSC (i, iv), UC-MSC (ii–v), and AT-MSC (iii, vi) stained for SM22α (i–iii) and α-SMA (iv–vi). Scale = 50 μm. All conditions n ≥ 3. (JPG 63 kb) [file 13287_2017_757_MOESM2_ESM.jpg]

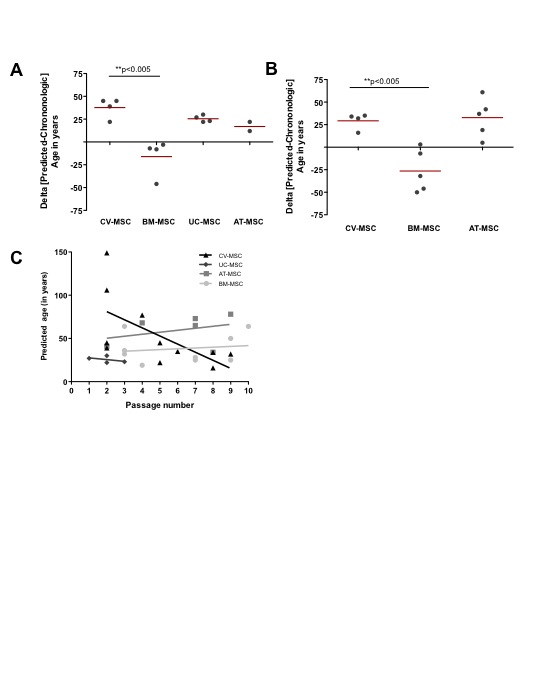

Supplement: Supplementary file 3 — A Difference between predicted and chronological MSC donor age (years) after EAS: CV-MSC 37.75 ± 5.43 years (n = 4), BM-MSC –16.00 ± 10.06 years (n = 4), UC-MSC 25.50 ± 1.84 years (n = 4), AT-MSC 17.00 ± 5.00 (n = 2), from passage 2 to passage 5 (**p < 0.005). B Difference between predicted and chronological MSC donor age (years) after EAS: CV-MSC 29.25 ± 4.46 years (n = 4), BM-MSC –26.40 ± 10.52 years (n = 5), AT-MSC 32.80 ± 9.65 (n = 5), from passage 6 to passage 15 (**p < 0.005). It was not possible to keep UC-MSC until late passages. C Predicted age (years) versus passage number EAS: one representative donor shown for CV-MSC (black), UC-MSC (dark gray), AT-MSC (medium gray), and BM-MSC (light gray). (JPG 33 kb) [file 13287_2017_757_MOESM3_ESM.jpg]

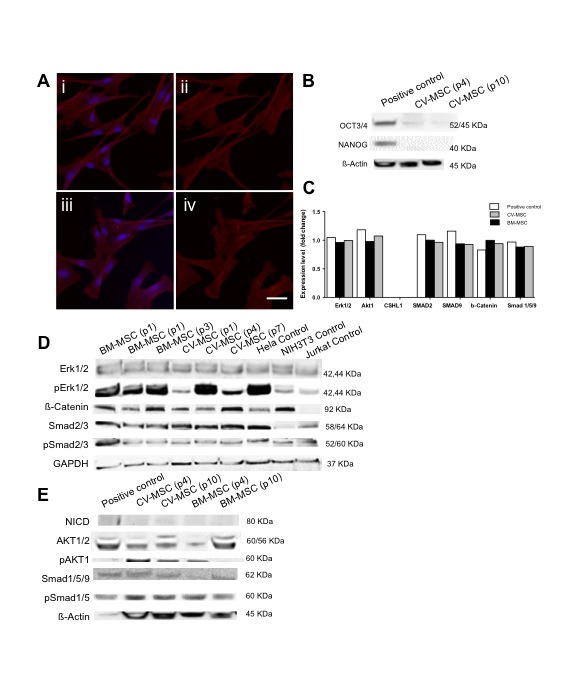

Supplement: Supplementary file 4 — A Immunofluorescence CV-MSC in passage 5 stained for Oct3/4 (i) and Nanog (ii). Alexa Fluor-488 (Green) was used to label the primary antibodies (i–iv). Rhodamine-TRITC was used for F-Actin fiber labeling (i–iv) and DAPI for nuclei counterstain (i, iii). Scale = 10 μm. B Western blot analysis for detection of Oct3/4 and Nanog proteins in passage 4 and passage 10 CV-MSC. hESC are positive control. C Relative ERK1/2, AKT1, CSHL1, SMAD2, SMAD9, β-CATENIN, and SMAD1/5/9 gene expression in CV-MSC in passage 4 and BM-MSC in passage 3. Data calibrated to positive control, expression of which is considered one for both genes. Housekeeping gene GAPDH used for normalization. D, E Western blot analysis to detect Erk1/2, pErk1/2, β-Catenin, Smad2/3, pSmad2/3, NICD, Akt1/2, pAkt, Smad1/5/9, and pSmad1/5 proteins in CV-MSC and BM-MSC. hESC, Hela, NIH3T3, and Jurkat controls included. (JPG 71 kb) [file 13287_2017_757_MOESM4_ESM.jpg]
